# Congenital Adrenal Hyperplasia (CAH) and Gitelman Syndrome (GS): Overlapping Symptoms in an Uncommon Association

**DOI:** 10.1155/2021/6633541

**Published:** 2021-03-08

**Authors:** Valeria Calcaterra, Giulia Roberto, Anna La Rocca, Beatrice Andrenacci, Federico Rossi, Gian Vincenzo Zuccotti, Valentina Fabiano

**Affiliations:** ^1^Pediatric and Adolescent Unit, Department of Internal Medicine, University of Pavia, 27100 Pavia, Italy; ^2^Department of Pediatrics, Children's Hospital “V. Buzzi”, 20157 Milano, Italy; ^3^Pediatric Unit, Fondazione IRCCS Policlinico S. Matteo and University of Pavia, 27100 Pavia, Italy; ^4^Department of Biomedical and Clinical Science “L. Sacco”, University of Milano, 20157 Milano, Italy

## Abstract

**Background:**

Classical salt-wasting (SW) congenital adrenal hyperplasia (CAH) and Gitelman syndrome (GS) are two genetic conditions in which dyselectrolytemia may occur. No association between the two conditions has been previously described. *Case Presentation*. We present the case of a boy with a neonatal diagnosis of SW-CAH who showed low potassium blood levels from the age of 15 years. This electrolytic alteration was, at first, attributed to an excessive action of mineralocorticoid drugs. Due to persistence of hypokalemia, SLC12A3 whole genome sequencing was performed, showing a heterozygous C to *T* base pair substitution at position 965 in gene SLC12A3. This mutation is related to Gitelman syndrome with autosomal recessive transmission.

**Conclusions:**

SW-CAH and GS determine opposite values of potassium in the absence of specific therapy, with a natural tendency to compensate each other. The symptom overlap makes diagnosis difficult. Organic causes of hypokalemia in patients undergoing life-saving therapy should not be excluded.

## 1. Introduction

Congenital adrenal hyperplasia (CAH) is a common autosomal recessive disorder characterized by impaired adrenal cortisol biosynthesis with associated androgen excess due to a deficiency of one or more enzymes in the steroidogenesis process within the adrenal cortex [1]. The severity and clinical features of CAH vary depending on the enzymatic defect, its residual activity, age of presentation, and genotype. The most common and prototypical example of the CAH disorders group (90–95%) is caused by 21-hydroxylase deficiency. This leads to defective cortisol synthesis, shunting of the accumulated steroid precursors through alternative pathways, and adrenal gland hyperplasia. 21-Hydroxylase deficiency (21-OHD) is classified into 3 subtypes according to clinical severity: classical salt wasting (SW), classical simple virilizing (SV), and nonclassical CAH (mild or late onset) [1]. Salt-wasting (SW) CAH is the most severe type, representing 75% of all cases of classical CAH; gluco- and mineralocorticoid deficiency occurs. If left untreated, low levels of aldosterone lead to hypovolemia, hyponatremia, hyperkalemia, hyperreninemia, development disorders, weight loss, convulsions, and finally death during the neonatal period (1–4 weeks after birth). Rapid diagnosis is crucial in order to increase the child's chances of survival. Treatment is based on replacement therapy with glucocorticoids and mineralocorticoids (with periodic monitoring of renin and aldosterone levels).

Gitelman syndrome (GS), also defined as familial hypokalemia-hypomagnesemia, is an inherited tubular disease resulting from mutations of the SLC12A3 gene encoding the thiazide-sensitive sodium-chloride cotransporter (NCC); it is responsible for NaCl reabsorption in the early distal convoluted tubules. The syndrome is transmitted as an autosomal recessive trait and is characterized by hypokalemic metabolic alkalosis, hypomagnesemia, and hypocalciuria with preserved kidney function [2].

The association between SW-CAH and GS has not been previously reported. We present the case of a Caucasian boy with an SW-CAH diagnosis, who showed low potassium blood levels from the age of 15 years, in which SLC12A3 mutation related to GS was detected.

### 1.1. Case Presentation

The patient was born to nonconsanguineous parents. In the first month of life, he was hospitalized for weight loss and dehydration. Blood analysis showed hyponatremia, hyperkalemia, and high androgenic hormones. Laboratory results at the time of diagnosis are given in Table 1. Therapy with hydrocortisone and fludrocortisone was therefore started immediately.

The southern blotting and PCR-based amplification analysis were used to detect a homozygous mutation in CYP21A2 gene [c.1066 C > *T* (pArg356Trp)]. The mutation was confirmed to be heterozygosis in both parents. A CAH diagnosis (OMIM 201910), subtype SW-CAH, was defined. The patient had regularly attended clinical check-ups, which showed normal auxological as well as pubertal development.

At the age of 15 years, diagnostic routine exams in his follow-up showed a low potassium blood level (2.7 mEq/L), which was confirmed by further testing. At first, this electrolytic alteration was attributed to excessive action of mineralocorticoid drugs, and thus, the fludrocortisone dose was reduced. Despite this modification, the blood levels of potassium remained below the normal range. Moreover, laboratory investigations revealed hypomagnesemia (1.3 mg/dl, normal values 1.7–2.5 mg/dl) and hypercalciuria. Nephrological evaluation was performed, excluding the glomerular origin of electrolytic alterations. Despite the persistent mild hypokalemia (range 2.6–3.1 mEq/L), the patient remained asymptomatic and no muscle symptoms or ECG alterations were ever referred.

Due to the persistence of hypokalemia, after obtaining informed consent from the patient, we required a next-generation sequencing in the suspicion of Gitelman syndrome. We found a heterozygous mutation in SLC12A*3* gene (variant c. [965C > *T*]; [ = ]p. [Ala322Val]; [ = ], NM_000339) described in the literature [3, 4] and in the human gene mutation database (access number CM117024) associated with Gitelman syndrome with an autosomal recessive transmission.

No replacement with electrolytic integration was prescribed since the patient remained asymptomatic. During monitoring until adult age, preserved kidney function with persistence of mild hypokalemia and hypomagnesemia was noted.

## 2. Discussion

CAH is a group of genetic conditions with a clinical heterogeneity [3, 5, 6]. The classical SW-CAH form appears when mutations in the CYP21A2 gene are so extensive that 21-hydroxylase loses nearly all its enzymatic activity (<2%) [3, 5–8]. SW-CAH results in life-threatening impairment of the synthesis of glucose and mineralocorticoids, and an early diagnosis is a key factor in saving a newborn's life [3]. The therapy should be individualized, adjusted, and tailored to minimize symptoms throughout the achievement of normal sexual development, fertility, height and aimed at a generally better quality of life. During adaptation of therapy in childhood, dyselectrolytemia may occur.

GS is an inherited autosomal recessive renal tubular disorder caused by loss-of-function mutations in the SLC12A3 gene. The SLC12A3 gene maps to chromosome region 16q13, consists of 26 exons, and encodes the renal thiazide-sensitive sodium chloride cotransporter (NCC), specifically expressed in the distal convoluted tubule [9]. The prevalence of GS ranges from 1/1000 to 9/10000. It is easily neglected due to mild clinical manifestations, including dyselectrolytemia, and good prognosis.

We are reporting the first case of CAH associated with GS. The dyselectrolytemia was initially attributed to an excess of mineralocorticoid action of fludrocortisone and subsequently diagnosed as GS. The patient presented a heterozygous mutation (C965 T) of the SLC12A3 gene, causing a substitution of alanine for valine at position 322. This variant was first reported by Vargas-Poussou et al. [3]; in the dbSNP (Single Nucleotide Polymorphism Database), the mutation is described as having a frequency in the European population of less than 1%. Although GS is an autosomal recessive disorder, homozygous mutations are found in only 18% of patients [10], more than 45% of GS cases have compound heterozygous mutations, 30% have single heterozygous mutations, and 7% have three or more mutations [11]. In the literature, no previous association between CAH and GS had been reported. In our case, comorbidity with CAH-SAW leads to an “atypical” compensation of potassium, and this probably accounts for the absence of classic GS signs (Table 2). The two pathologies determined the opposite values of potassium (hyperkalemia in CAH and hypokalemia in GS) in the absence of specific therapy with a natural tendency to compensate each other.

This case prompted us to seriously consider other possible, albeit less frequent, organic causes of hypokalemia in patients undergoing life-saving therapy.

In conclusion, in patients with CAH undergoing specific therapy, it is important not to forget other possible but less frequent organic causes of hypokalemia, in order to establish the appropriate treatment. The symptom overlap makes diagnosis difficult.

## Figures and Tables

**Table 1:** Laboratory results of our patient at time of SW-CAH syndrome diagnosis.

Parameter (unit)	Result	Normal range for age
Serum sodium (mEq/L)	129	135–153 mEq/L
Serum potassium (mEq/L)	5.7	3.5–5.3 mEq/L
17*α*-Hydroxyprogesterone	266	0.49–2.3 ng/ml
Aldosterone	>1000	70–300 pg/ml
Renina	>20	3–33 pg/ml

**Table 2:** Signs in typical GS cases and our patient.

Signs	Gitelman	Our patient
Growth retardation	±	−
Tetany	+	−
Hypokalemic alkalosis	+	−
Hypomagnesemia	+	+
Chondrocalcinosis	+	−
